# Special Notice

**Published:** 1899-07

**Authors:** 


### SPECIAL NOTICE.

Will our correspondents kindly bear in mind that all matter intended for publication in this journal, outside of the advertising department, should be sent direct to the editor, Dr. James Truman, 4505 Chester Avenue, Philadelphia, while all matter pertaining to the business must be addressed to J. B. Lippincott Company, 718

Filbert Street, Philadelphia. Inquiries relating to advertisements should be sent to H. T. Pearce, 1914 Cherry Street, Philadelphia. All checks should *be drawn to the order* of J. B. Lippincott Company. Attention to these requirements will be greatly appreciated by those having charge of the several departments of the INTERNATIONAL DENTAL JOURNAL.

